# Olfactory Stimulation Effect of Aldehydes, Nonanal, and Decanal on the Human Electroencephalographic Activity, According to Nostril Variation

**DOI:** 10.3390/biomedicines7030057

**Published:** 2019-07-31

**Authors:** Minju Kim, Kandhasamy Sowndhararajan, Hae Jin Choi, Se Jin Park, Songmun Kim

**Affiliations:** 1 School of Natural Resources and Environmental Science, Kangwon National University, Chuncheon 24341, Korea; 2 Department of Botany, Kongunadu Arts and Science College, Coimbatore 641029, India; 3 Foundation of Agri. Ech. Commercialization & Transfer (FACT), Iksan 54667, Korea

**Keywords:** electroencephalography, decanal, inhalation, nonanal, nostril

## Abstract

Fragrances play a pivotal role in humans’ psychological and physiological functions through the olfactory system. Aldehydes are important organic compounds with a variety of fragrance notes. Particularly, nonanal (C9) and decanal (C10) aldehydes are important natural fragrant components used to enhance floral, as well as citrus notes in perfumery products. In general, each nostril of the human nose is tuned to smell certain odor molecules better than others due to slight turbinate swelling between the nostrils. Hence, the objective of the present investigation was aimed to evaluate the influence of binasal and uninasal inhalations of C9 and C10 aldehydes on human electroencephalographic (EEG) activity. Twenty healthy participants (10 males and 10 females) participated in this study. The EEG readings were recorded from 8 electrodes (QEEG-8 system) according to the International 10-20 System. The results revealed that C10 exposure exhibited significantly different EEG changes, during binasal and uninasal inhalations. In different brain regions, C10 odor markedly decreased the absolute alpha and absolute beta power spectra. In regards to C9 odor, significant changes of EEG power spectra were noticed only during binasal inhalation. In addition, C10 mainly produced changes at the left parietal site (P3) than other brain sites. In conclusion, the variations in EEG activities of C9 and C10 aldehydes might be owing to their characteristic fragrance quality, as well as the influence of nostril differences.

## 1. Introduction

The human nasal cavity is a primary sensory organ, which contains a couple of nerve systems such as the trigeminal system and the olfactory system [1,2]. In the olfactory system, fragrances may come into contact with olfactory sensory neurons through retronasal and orthonasal pathways. The retronasal olfaction happens when fragrances enter into the nasal cavity through the mouth via the back of the nose. Whereas, the orthonasal olfaction occurs when fragrances are inhaled through the nose by smelling or sniffing [3]. The size of the left and right nostrils also varies remarkably among the individuals, due to an uneven septum. Consequently, airflow is greater in one nostril than in the other, and the variations in olfactory processing are highly associated with the nasal cycle. Hence, each nostril of the human nose is regulated to smell some odors better than others [4,5]. Therefore, bi-nasal or uni-nasal inhalation of fragrances may affect the functions of the brain differently. 

Fragrances play a vital role in the psychophysiological activities of the human brain [6,7]. In perfumery industries, volatile compounds are considered as main sources of natural fragrances. The functional changes of the brain, provoked through exposure to fragrances, are highly connected with the alteration of neuronal electrical activity [8,9]. Previously, several studies have reported that electroencephalograph (EEG) techniques are commonly used for determining spontaneous brain wave alterations during the inhalation of fragrances [10,11,12,13]. In a recent study, the inhalation of *Abies koreana* essential oil, through both nostrils and uninostrils, exhibited significantly different EEG activities in different brain regions [14].

Aldehydes are important organic compounds that incorporate a carbonyl functional group (C=O). Aldehydes have been used to enhance a range of fragrance notes, such as metallic, starchy and citrusy to green, fatty, and waxy. Aldehydes are derived from various sources in nature, such as plants, microbes, and animals [15]. In the legendary perfume, Chanel No. 5, aldehydes form the primary floral scent compounds, due to their pleasant smell. Aldehydes, especially octanal (C8), no-nanal (C9), and decanal (C10), which are widely used fragrant compounds in perfumery products for green-floral and aldehydic fragrance types [16]. 

In these, C9 and C10 aldehydes are widely used fragrant components in perfume industries. C9 aldehyde is commonly used to enhance rose-like fragrance notes in various perfumery products, and is naturally found in numerous essential oils, including cinnamon, lemongrass, milkweed, citrus, and rose [17,18]. Further, C9 is one of the important skin odorants of birds and humans, resulting from the oxidation of sebaceous fatty acids [19]. Another aldehyde, C10 has characteristic odors of waxy, aldehydic, orange, and citrus. C10 aldehyde is predominantly used for floral and citrus like scents in various perfumery products. The aldehyde, C10 is an important component in a variety of essential oils, such as neroli oil, kesum oil, coriander oil, different citrus peel oils, and many Mediterranean woody species. It is one of the main compounds of buckwheat odor [20,21]. With this background, the aim of the present study was to evaluate the effect of exposure of C9 and C10 aldehydes on human EEG activity, in order to understand the influence of binasal and uninasal inhalations.

## 2. Methods

### 2.1. Materials

Nonanal (CAS No. 124-19-6) and decanal (CAS No. 112-31-2) were purchased from Sigma (St. Louis, MO, USA). These chemicals were kept at 4 °C until used for electroencephalographic (EEG) study. 

### 2.2. Subjects

Twenty healthy volunteers (10 males and 10 females) aged 20–30 years participated in the EEG measurements. All participants were university students. All participants were non-smokers and right-handed without any defects in their olfaction. All the subjects provided informed consent before participation.

### 2.3. Experimental Design

The one-group pre-test and post-test research design was used in this study (20 participants). The participants were informed that the objective of the study was to investigate the effects of exposure of fragrant compounds on EEG activity. During EEG measurements, the participants were told to sit quietly without any movement, keep their eyes fully closed, and breathe naturally, but to remain conscious throughout. After the EEG measurements, the participants were asked to provide their preference and impressions of the C9 and C10 fragrances.

### 2.4. EEG Recordings

A QEEG-8 system was used to record the EEG readings (LXE3208, LAXTHA Inc., Daejeon, Republic of Korea). The EEG data were recorded from 8 channels placed on the scalp, according to the International 10–20 System previously described by Kim et al. [10]. The electrodes were referenced to the ipsilateral earlobe electrodes. The EEG sampling rate of the measured subjects was 256 Hz, filtered in the range of 0.5–50 Hz, and the readings were stored in a computer by the 12-bit AD conversion. The ECI electrode gel was applied into each electrode to connect with the surface of the scalp in order to drop the electric resistance of the scalp below 5 kΩ. To remove noise, a 1–1000 Hz band pass filter was employed with a 24 dB/octave roll-off, and a 60 Hz notch filter was applied.

### 2.5. Fragrance Administration

The aldehydes, C9 and C10 were used as the fragrance stimuli. The EEG measurement room was maintained at a constant temperature of 23 °C with a relative humidity of 50%. The undiluted fragrance stimulus (10 μL) was dropped on one edge of the perfumer’s paper strip and then placed approximately 30 mm in front of the nose of subjects. EEG was recorded 45s during no odor and 45s during the exposure of C9 and C10. EEG data were recorded separately for binasal, as well as uninasal inhalations during no odor and C9 or C10 odor exposure. During the uninasal EEG recordings, one nostril was completely blocked by using the cotton. The interval time between each condition [binasal and uninasal (left and right nostrils)] was 3 min. First, the EEG was recorded during no odor and C9 odor exposure. Then the EEG was recorded during no odor and C10 odor exposure. 

### 2.6. Data Analysis

Two EEG segments were selected for each condition, 45 s for no odor presentation and 45 s for odor presentation. The Fast Fourier transform was used to measure the mean power values (μV^2^) of each segment. The mean power spectrum values were estimated for 25 EEG indices such as AT, Absolute theta (4~8 Hz); AA, Absolute alpha (8~13 Hz); AB, Absolute beta (13~30 Hz); AG, absolute gamma (30~50 Hz); ASA, Absolute slow alpha (8~11 Hz); AFA, Absolute fast alpha (11~13 Hz); ALB, Absolute low beta (12~15 Hz); AMB, Absolute mid beta (15~20 Hz); AHB, Absolute high beta (20~30 Hz); RT, Relative theta (4~8 Hz)/(4~50 Hz); RA, Relative alpha (8~13 Hz)/(4~50 Hz); RB, Relative beta (13~30 Hz)/(4~50 Hz); RG, Relative gamma (30~50 Hz)/(4~50 Hz); RSA, Relative slow alpha (8~11 Hz)/(4~50 Hz); RFA, Relative fast alpha (11~13 Hz)/(4~50 Hz); RLB, Relative low beta (12~15 Hz)/(4~50 Hz); RMB, Relative mid beta (15~20 Hz)/(4~50 Hz); RHB, Relative high beta (20~30 Hz)/(4~50 Hz); RST, Ratio of SMR to theta (12~15 Hz)/(4~8 Hz); RMT, Ratio of mid beta to theta (15~20 Hz)/(4~8 Hz); RSMT, Ratio of SMR~mid beta to theta (12~20 Hz)/(4~8 Hz); RAHB, Ratio of alpha to high beta (8~13 Hz)/(20~30 Hz); SEF50, Spectral edge frequency 50% (4~50 Hz); SEF90, Spectral edge frequency 90% (4~50 Hz); ASEF, Spectral edge frequency 50% of alpha (8~13 Hz) [13]. Statistical analysis was performed with SPSS version 18 statistics for windows (SPSS, Inc., Chicago, IL, USA). The values of EEG power spectra during no odor, and during the exposure of C9 or C10 odor, were analyzed by a paired Student’s *t*-test. The *p* value < 0.05 was considered statistically significant. EEG data batch processing and the brain 3D-mapping of EEG power spectra were performed using Telescan software (LXSMD61, LAXTHA Inc., Daejeon, Republic of Korea).

## 3. Results

**Figure 1:**
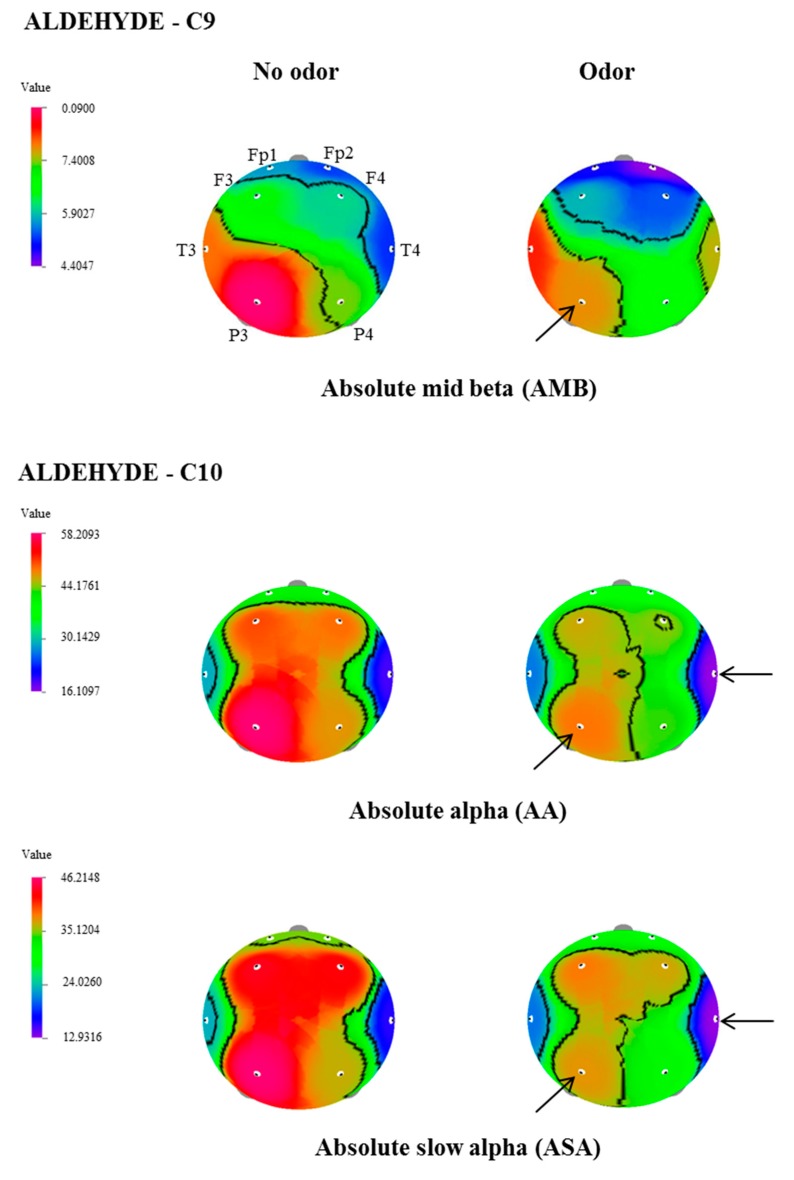
The t-Mapping of electroencephalographic (EEG) power spectrum changes during the binasal inhalation of aldehydes (C9 and C10). Fp1, left prefrontal; Fp2, right prefrontal; F3, left frontal; F4, right frontal; T3, left temporal; T4, right temporal; P3, left parietal; P4, right parietal. Arrows show significant changes in the regions during the inhalation of C9 and C10.

**Figure 2:**
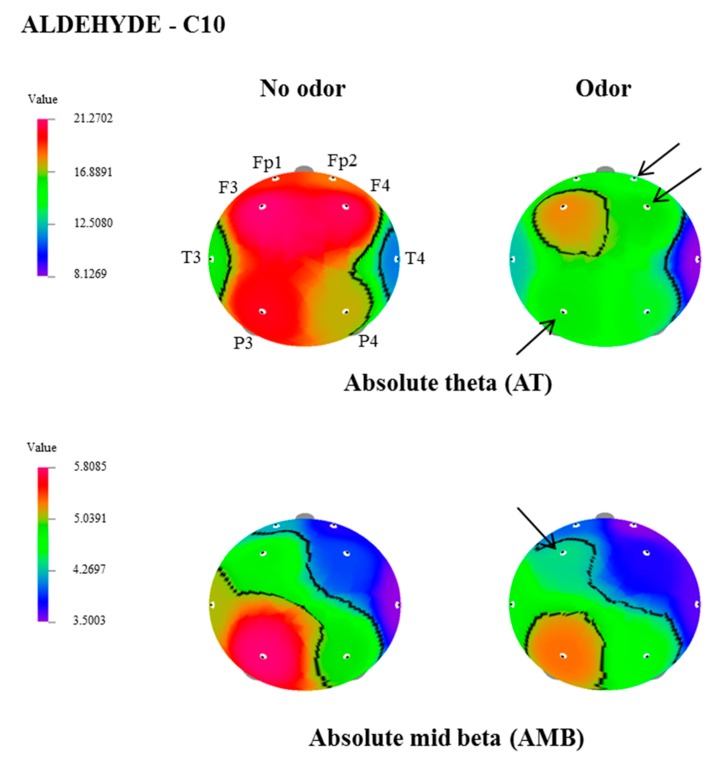
The t-Mapping of EEG power spectrum changes during the left nostril inhalation of C10 aldehyde. Fp1, left prefrontal; Fp2, right prefrontal; F3, left frontal; F4, right frontal; T3, left temporal; T4, right temporal; P3, left parietal; P4, right parietal. Arrows show significant changes in the regions during the inhalation of C10.

**Figure 3:**
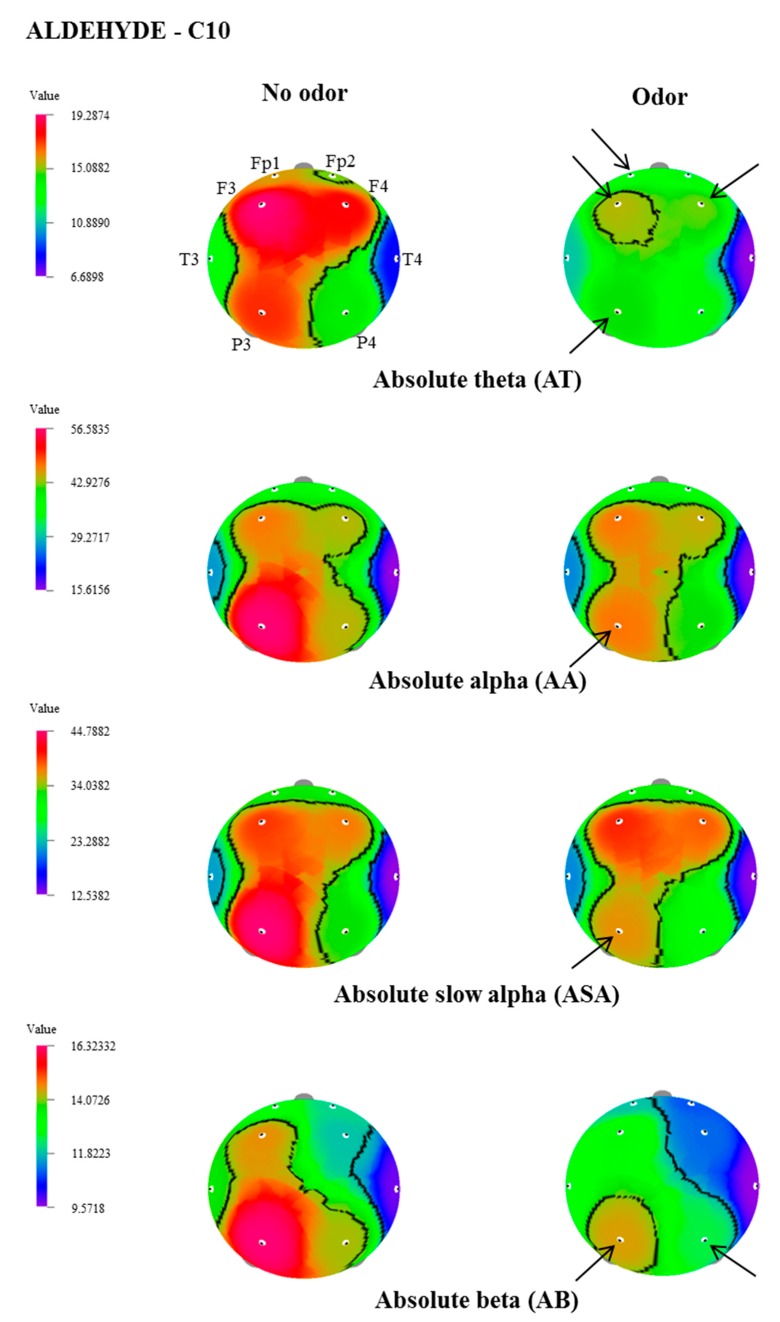
The t-Mapping of EEG power spectrum changes during the right nostril inhalation of C10 aldehyde. Fp1, left prefrontal; Fp2, right prefrontal; F3, left frontal; F4, right frontal; T3, left temporal; T4, right temporal; P3, left parietal; P4, right parietal. Arrows show significant changes in the regions during the inhalation of C10.

**Figure 4:**
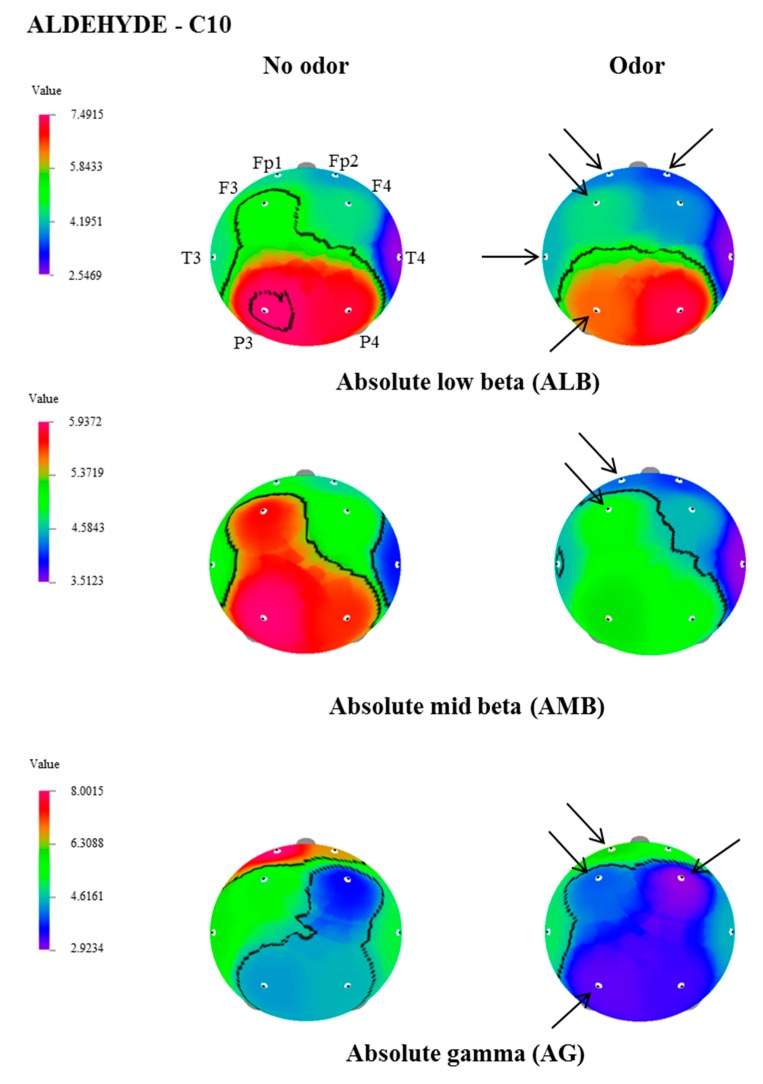
The t-Mapping of EEG power spectrum changes during the right nostril inhalation of C10 aldehyde. Fp1, left prefrontal; Fp2, right prefrontal; F3, left frontal; F4, right frontal; T3, left temporal; T4, right temporal; P3, left parietal; P4, right parietal. Arrows show significant changes in the regions during the inhalation of C10.

In this study, the effect of inhalation of undiluted aldehydes, C9 and C10, on EEG activity in relation to nostril variation, was investigated. The changes of EEG power spectrum values, during no odor, and C9 or C10 odor conditions, were analyzed from 8 electrode sites such as Fp1, Fp2, F3, F4, T3, T4, P3, and P4. Table 1, Table 2 and Table 3 show EEG power spectra changes due to the exposure of C9 and C10 via both nostrils, left nostril, and right nostril. The t-mapping of brain wave changes clearly expressed the modification of EEG waves during no odor and during the exposure of C9 and C10 odors (Figure 1, Figure 2, Figure 3 and Figure 4). The results revealed that the binasal and uninasal inhalations showed significantly different EEG activity. 

In the EEG analyzes, a significant change (*p* < 0.05) of absolute wave activity was noticed in only one index, due to the exposure of C9 via binasal. Whereas significant changes of 2 indices during binasal inhalation, 2 indices during left nostril inhalation, and 7 indices during right nostril inhalation were observed for C10. In the binasal inhalation, absolute mid beta value significantly declined at the P3 site (8.988–7.7534 μV^2^) during the inhalation of C9. However, C9 odor did not show any significant variation in the absolute wave activity via the left nostril, as well as the right nostril inhalations. In regards to C10, absolute alpha and slow alpha waves significantly decreased at T4 (18.9484–16.1097 μV^2^, and 58.2094–48.7095 μV^2^, respectively) and P3 (15.9347–12.9317 μV^2^ and 46.2148–37.5394 μV^2^) regions, due to the exposure of C10. 

In the left nostril inhalation, absolute theta at Fp1, F4, and P3 regions, and absolute mid beta at the F3 region, significantly decreased. due to the exposure of C10, when compared with no odor exposure. In the case of right nostril inhalation, significant decreases of absolute theta (Fp1, F3, F4 and P3), absolute alpha (P3), absolute slow alpha (P3), absolute beta (P3 and P4), absolute gamma (Fp1, F3, F4 and P3), absolute low beta (Fp1 and F3) and absolute mid beta (Fp1, Fp2, F3, T3 and P3) were observed during the exposure of C10. The results revealed that the binasal and uninasal inhalations of C10 produced EEG power spectra changes mainly at the P3 region.

## 4. Discussion

In recent times, a number of studies have reported the influence of fragrances on psychological and physiological conditions of human [6,7]. In addition, there have been common interests in the perceptual interaction concerning both sides of the ears, eyes, and vestibular apparatuses. But, less concern has been shown to the interaction among the two nostrils of the nose [14,22]. For this purpose, the EEG is a widely used electrophysiological method to determine the effect of exposure of odors on brain function. Therefore, we studied the olfactory stimulation effect of aldehydes, C9 and C10 on human EEG activity according to the nostril difference. 

In this study, significant decreases of most of the absolute waves were noticed in different regions with the exception of absolute fast alpha and absolute high beta waves (Table 1, Table 2 and Table 3). Previous studies clearly suggest that the alpha wave activity is diminished during emotional tension and stress conditions [23,24,25]. Hence, the decrease in alpha wave activity, due to the exposure of C10, may be associated with the tension and stress states of the brain. In general, the reduction in beta wave activity is mainly related to the drowsiness state, and an increase in beta wave activity is highly associated with alertness or attention states of the brain [26]. Furthermore, a significant reduction of theta activity was noticed, due to the exposure of C10 via the left and right nostril inhalations. The reduction in theta wave activity is mainly linked to the formation of memory [13,27,28]. In the present study, the reduction of theta wave activity suggests that the binasal and uninasal inhalations of C10 may enhance memory formation. However, AA, ASA, AB, AG, ALB, and AMB activities significantly decreased, due to C10 exposure. Hence, the inhalation of C10 may increase the stress, tension, and drowsiness states of the brain. Furthermore, the findings of the present study indicate that the fragrance inhalation of C10 mainly produced changes at the left parietal region than other regions. 

**Figure 5:**
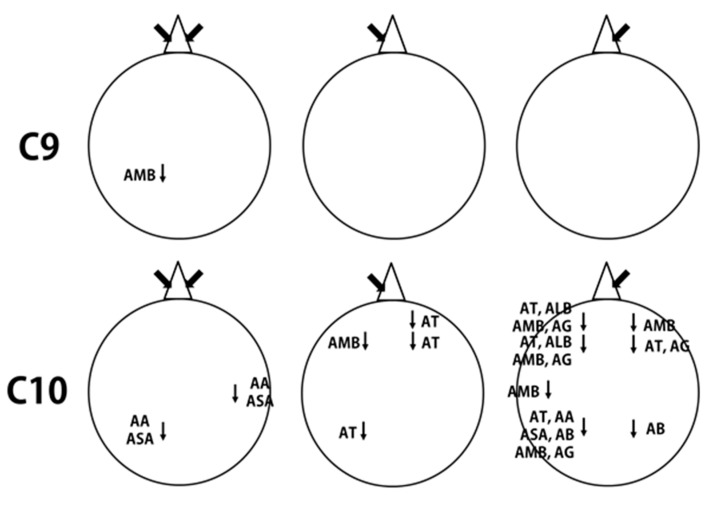
A schematic representation of the EEG power spectrum changes during the binasal, left nostril and right nostril inhalations of aldehydes, C9 and C10. AT, absolute theta; AA, absolute alpha; ASA, absolute slow alpha; AB, absolute beta; AMB, absolute mid beta; ALB, absolute low beta; AG, absolute gamma.

The binasal and uninasal inhalations of C9 and C10 showed different EEG power spectrum changes (Figure 5). The differences in the EEG activities of aldehydes, between the left and right nostril inhalations, may be due to the slight turbinate swelling in any one of the nostrils. Hence, each nostril receives fragrances at different rates during inhalation and sends a slightly different electrical signal to the brain [5,22]. Searleman et al. [29] suggested that the stimulation of the right and left nostrils mainly affect the right, and left hemispheres, respectively. Further, the nasal cycle plays an important role in the identification of fragrances. The naming of fragrances was more accurate when fragrances were presented through the left nostril, when compared to the right nostril [30]. Gudziol, Paech, and Hummel [31] observed that 15% of the healthy subjects exhibited nostril side differences in the identification of fragrances. The authors also reported that older persons exhibited larger nostril side differences during the identification of fragrances than younger persons.

In the slow yogic breathing, Pal et al. [32] found that left nostril may relieve stress and decrease the risks of cardiovascular disease, when compared with right nostril breathing. In our recent study, the exposure of essential oil of *Abies koreana* twigs, via binasal and uninasal, significantly produced different EEG activities. In the binasal inhalation, significant increases of absolute alpha at the left frontal and right parietal sites, and absolute fast alpha at the right parietal sites, were observed. On the other hand, absolute beta and theta activity significantly decreased (F4, P3, and P4) during the uninasal inhalation [14].

In general, C9 aldehyde has a rose-like smell, whereas C10 aldehyde has floral with citrus-like smell. These aldehydes exhibit different EEG activities as a result of the dis-similarity in their selectivity, as well as the sensitivity of odorant receptors. Regarding the location of EEG changes, both the components significantly affect the left parietal site (P3) than other brain sites. The parietal region has been mainly associated with the integration of sensory information from different parts of the human body, especially in their recognition and perception of stimuli [7,33]. The sensitivity of the parietal region may also be responsible for variations in the EEG activity, due to the exposure of C9 and C10 odors. Overall, the results revealed that right nostril inhalation produced significant changes in more numbers of EEG indices, than left nostril and binasal inhalations. The results of the present investigation clearly suggest that these aldehydes significantly produced changes in different EEG power spectra at different brain sites according to the nostril. 

The findings of the present study revealed that C9 and C10 aldehydes significantly decreased the absolute waves in different regions. In particular, the decrease in absolute alpha and beta waves are not associated with the positive psychophysiological conditions of the human. Furthermore, nostril differences play a key role in the EEG activity of C9 and C10 aldehydes. It is concluded that the variation in the EEG activity of C9 and C10 aldehydes are mainly related to the fragrance types, as well as nostril differences. 

The present study has some limitations. In this study, undiluted C9 and C10 compounds were used as fragrance stimuli; the results may have differed if the concentration of these compounds was changed. Further, the EEG readings were recorded for a short duration (45 s for no odor, and 45 s for odor conditions). In addition, there have been significant variations in the EEG activity according to gender. In light of these limitations, EEG recordings for a slightly longer duration, and different concentrations of C9 and C10, are needed for understanding their exact action on EEG oscillations.

## Figures and Tables

**Table 1:** Significant changes of electroencephalographic (EEG) power spectrum values during the binasal inhalations of aldehydes (C9 and C10).

Aldehyde	EEG Indices	Site	No Odor Exposure (µV^2^)	Odor Exposure (µV^2^)	*t*-Test	*p* Value *
C9	AMB	P3	8.8988 ± 1.3	7.7534 ± 1.4	2.119	0.047
C10	AA	T4	18.9454 ± 3.2	16.1097 ± 2.7	2.332	0.031
		P3	58.2094 ± 10.9	48.7095 ± 8.2	2.356	0.029
	ASA	T4	15.9347 ± 3.1	12.9317 ± 2.62	2.836	0.011
		P3	46.2148 ± 9.6	37.5394 ± 7.5	2.458	0.024

AA, absolute alpha; ASA, absolute slow alpha; AMB, absolute mid beta * Significant difference (*p* < 0.05), Number of subjects—20.

**Table 2:** Significant changes of EEG power spectrum values during the left nostril inhalation of C10 odor.

EEG Indices	Site	No Odor Exposure (µV^2^)	Odor Exposure (µV^2^)	*t*-Test	*p* Value *
AT	Fp2	18.3174 ± 2.7	14.0908 ± 1.7	2.130	0.046
	F4	20.8985 ± 2.8	16.3078 ± 1.9	2.327	0.031
	P3	20.1413 ± 2.7	16.0885 ± 2.2	2.240	0.037
AMB	F3	4.8342 ± 0.4	4.3673 ± 0.3	2.307	0.032

AT, absolute theta; AMB, absolute mid beta. * Significant difference (*p* < 0.05), Number of subjects—20.

**Table 3:** Significant changes of EEG power spectrum values during the right nostril inhalation of C10 odor.

EEG Indices	Site	No Odor Exposure (µV^2^)	Odor Exposure (µV^2^)	*t*-Test	*p* Value *
AT	Fp1	15.8579 ± 2.6	13.2670 ± 1.7	2.153	0.044
	F3	19.2875 ± 3.2	15.4492 ± 2.0	2.649	0.016
	F4	18.0308 ± 3.4	14.9039 ± 2.2	2.113	0.048
	P3	17.3454 ± 2.8	14.5731 ± 1.9	2.363	0.029
AA	P3	56.5838 ± 9.7	47.2791 ± 8.3	2.318	0.032
ASA	P3	44.8242 ± 8.9	36.4876 ± 7.6	2.273	0.035
AB	P3	16.3236 ± 1.8	14.4824 ± 1.6	3.216	0.005
	P4	14.2120 ± 1.5	12.4604 ± 1.2	3.049	0.007
ALB	Fp1	4.3595 ± 0.6	3.7719 ± 0.5	2.367	0.029
	F3	5.1939 ± 0.7	4.4375 ± 0.5	2.213	0.039
AMB	Fp1	4.4511± 0.4	3.7999 ± 0.3	3.142	0.005
	Fp2	4.0911 ± 0.4	3.6095 ± 0.3	2.380	0.028
	F3	5.3220 ± 0.6	4.5441 ± 0.4	2.231	0.038
	T3	4.8826 ± 0.5	4.3729 ± 0.5	2.541	0.020
	P3	5.8663± 0.7	5.2261 ± 0.6	2.409	0.026
AG	Fp1	8.0015 ± 1.0	6.1253 ± 0.8	2.821	0.011
	F3	5.1881 ± 0.7	4.0128 ± 0.5	2.493	0.022
	F4	3.5508 ± 0.6	2.9234 ± 0.4	2.188	0.041
	P3	4.3676 ± 0.7	3.1706 ± 0.4	2.204	0.040

AT, absolute theta; AA, absolute alpha; ASA, absolute slow alpha; AB, absolute beta; AMB, absolute mid beta; ALB, absolute low beta; AG, absolute gamma; * Significant difference (*p* < 0.05), Number of subjects—20.
